# Complete Genome Sequence of a Hemagglutination-Negative Avian Paramyxovirus Type 4 Isolated from China

**DOI:** 10.1128/genomeA.00045-13

**Published:** 2013-03-14

**Authors:** Kai-Cheng Wang, Gui-Qian Chen, Wen-Ming Jiang, Shuo Liu, Guang-Yu Hou, Jian-Min Yu, Jin-Ping Li, Qing-Ye Zhuang, Ji-Ming Chen

**Affiliations:** China Animal Health and Epidemiology Center, Qingdao, People’s Republic of China

## Abstract

We report here the complete genome sequence of a nonpathogenic and hemagglutination-negative avian paramyxovirus type 4 isolated from a duck in southern China. Phylogenetic analysis of the genome sequence indicated that the waterfowl virus possibly has evolved into the Eastern and Western Hemisphere lineages.

## GENOME ANNOUNCEMENT

Avian paramyxoviruses (APMVs) belong to the genus *Avulavirus* in the family *Paramyxoviridae*. So far, 11 serological types of APMVs, from APMV-1 (the well-characterized Newcastle disease virus) to APMV-11, have been described (1–7). Among them, APMV-4 has been reported to cause mild respiratory pathology in experimental chickens (2, 8). To date, five whole and 24 partial genomic sequences of only 25 APMV-4 isolates have been reported to GenBank. All 25 viruses were from wild or domestic waterfowls, with one from North America, one from Africa, seven from Europe, and 15 from Asia (2–7). It is believed that nearly all APMVs, including APMV-4 but with the exception of APMV-5, hemagglutinate chicken red blood cells (RBCs) (8).

We isolated a strain of APMV-4, APMV4/duck/China/G302/2012, from the feces of an apparently healthy duck in southern China in 2012 using embryonated chicken eggs. Surprisingly, the isolate did not hemagglutinate the RBCs of chickens, ducks, geese, and humans (type O), possibly due to some mutations in the viral hemagglutinin-neuraminidase (HN) gene. The isolate did not cause any clinical signs in 10 specific-pathogen-free chickens within 10 days following intravenous inoculation with 0.2 ml of a 1:10 dilution of bacterium-free infective allantoic fluid.

We obtained the genome sequence of the isolate through next-generation sequencing using the Ion Torrent personal genome machine (PGM, Life Technologies) and a novel RNA-Seq method which will be reported separately. The genome is 15,054 nucleotides (nt) in length, which is consistent with the “rule of six” (8). The genome contains six nonoverlapping genes in the order 3′-NP–P/V/W-M-F–HN-L-5′, which is typical in APMVs with the exception of APMV-6 (8). Theoretical amino acid lengths of the eight putative proteins encoded by the genome are as follows: nucleoprotein (NP), 457 amino acids (aa); V, W, and P, 274 aa, 137 aa, and 393 aa, respectively; matrix (M), 370 aa; fusion (F), 566 aa; HN, 565 aa; and L, 2,211 aa. The genes are flanked on either side by highly conserved transcription start and stop signals and have intergenic sequences ranging in length from 9 to 42 nt. The genome contains a 55-nt leader region at the 3′ end and a 17-nt trailer region at the 5′ end.

Phylogenetic analysis and genetic distances were calculated by the neighbor-joining method under the Kimura 2-parameter model using the Molecular Evolutionary Genetics Analysis (MEGA) 5.03 software (9). Bootstrap values were calculated out of 1,000 replicates. Rates among the sites were set under gamma distribution, and the gamma parameter was set at 4.0. The results suggest that this waterfowl virus, similar to some subtypes of avian influenza viruses (10), has possibly evolved into two lineages: the Western Hemisphere lineage, represented by AMPV4/duck/Delaware/549227/2010, and the Eastern Hemisphere lineage, covering all the isolates from Asia, Europe, and Africa. Genetic distances within the lineages were <10.1%, while genetic distances between the lineages were >15.4% and have increased during the past few decades.

### Nucleotide sequence accession number.

The complete genome sequence of the strain APMV4/duck/China/G302/2012 has been submitted to GenBank under the accession no. KC439346.
